# Barriers to Infectious Disease Care among Lesbians^1^

**DOI:** 10.3201/eid1011.040467

**Published:** 2004-11

**Authors:** Jeanne M. Marrazzo

**Affiliations:** * University of Washington, Seattle, Washington, USA

**Keywords:** lesbian, bisexual, homosexuality, infectious disease, access, screening, Pap smear, sexually transmitted disease

## Abstract

Despite the considerable number of women in the United States who identify as lesbian, few data exist that address lesbians' health needs. The Institute of Medicine emphasized that data on sexually transmitted infections, Pap smear screening, and cervical dysplasia among lesbians were needed to guide clinical practice, policy development, and patient education. Use of surveillance data for this purpose is limited because risk classifications exclude same-gender sex among women or subsume it under behavior considered as higher risk. However, sexual transmission of human papillomavirus, HIV, *Treponema pallidum*, and *Trichomonas vaginalis* between women has been reported. Data indicate that lesbians receive routine Pap smear screening less frequently than is optimal. Moreover, lesbians commonly report previous pregnancy, induced abortion, and hormonal contraceptive use. Education of lesbians and their care providers should counter assumptions that sex between women confers no risk for transmission of sexually transmitted infections, and lesbians should receive Pap smears according to current guidelines.

A causative link between a patient's sexual orientation and access to healthcare is not evident. Why should identification as a lesbian or practice of same-sex behavior affect a patient's access to infectious diseases–related care? We discuss data to support this connection and make appropriate recommendations. Because this article discusses a subset of women defined by their practice of sex with other women, it will focus primarily on sexually transmitted infections (STIs) and their consequences. For the sake of simplicity, we will use the term "lesbian" to refer to a woman who engages in sex with another woman, and thus represents the axis of sexual behavior, which may not necessarily be congruent with self-defined sexual identity (*1*).

In the United States, estimates of lifetime same-gender sexual behavior among women are 8% to 20%, and 1.4%–4.3% of all women may currently be sexually active with other women (*1**,**2*). An estimated 2.3 million women specifically describe themselves as lesbian (*3*). Despite these considerable numbers, relatively little data are available on important health outcomes for these women, including prevalence of STI, HIV, and cervical cancer. Until recently, the major national women's health studies did not collect information on same-sex behavior or sexual identity (*4*). In its 1999 report, Lesbian Health: Current Assessment and Directions for the Future, the Institute of Medicine emphasized that more data were needed on STIs, Pap smear screening, and risk for cervical cancer in lesbians (*3*).

## Sexual Behavior and STIs

Attempts to use national or local surveillance data to estimate the risk for STI transmission between women are limited by the fact that many risk classification schemes have either excluded same-gender sex among women or subsumed it under a hierarchy of other behaviors viewed as higher risk. Moreover, few, if any, state or local STI reporting systems routinely collect information on same-sex behavior among women. The available data are derived from two sources: small studies that have directly measured prevalence of common STIs, usually among clinic attendees or self-referred study volunteers, and surveys that have queried lesbians about their self-reported STI history. Many of these studies have also assessed lesbians'self-report of sexual practices. Taken as a whole, these data indicate that the risk for STI transmission between women depends on the specific STI under consideration, and the sexual practices involved.

Some sexual practices, including oral-genital sex, vaginal or anal sex using hands, fingers, or penetrative sex toys, and oral-anal sex, are commonly practiced by female sex partners (*5**–**7*). Practices involving digital-vaginal or digital-anal contact, particularly with shared penetrative sex toys, present a plausible means for transmission of infected cervicovaginal secretions. This concept is most directly supported by reports of metronidazole-resistant trichomoniasis and genotype-concordant HIV, which have been sexually transmitted between women who reported these behaviors (*8**,**9*). Reports of *Chlamydia trachomatis* and *Neisseria gonorrhoeae* transmission between women are anecdotal and largely unpublished; however, approximately 3%–5% of respondents to surveys assessing lesbians' lifetime history of these STIs indicate that a chlamydial infection had been diagnosed by a healthcare provider. Among 6,146 respondents in the National Lesbian and Bi Women's Health Survey, conducted in the early 1990s, women reported contracting an STI from a female partner (including herpes by 135 persons, chlamydia by 102, genital warts by 100, gonorrhea by 16, hepatitis by 9, and HIV by 1) (*10*). Although self-report of STI history is often inaccurate (as is attribution of STI to a specific source partner), these data indicate that the respondents sought care for perceived genitourinary abnormalities and received a diagnosis that indicated the provider had reason to suspect an STI. The most important bacterial STI for which more precise data are needed is *C. trachomatis* infection. Although the rate of transmission of STIs between women is probably low relative to that of transmission from men to women, a substantial proportion of lesbians, including those who self-identify as lesbians, and especially younger women most at risk for chlamydial infection, may continue to have sex with men (*11*).

Transmission of common viral STIs, especially human papillomavirus (HPV) and herpes simplex virus (HSV) infections and of *Treponema pallidum*, the causative agent of syphilis, requires only skin-to-skin or mucosa contact, which can easily occur in the context of lesbian sex. Equally important, most lesbians (53%–99%) have had sex with men, and many (21%–30%) continue to do so (*11*); they may acquire viral STIs from men and subsequently transmit them to female partners. In addition to case reports, two studies have detected HPV DNA by PCR-based methods in 13% to 30% of lesbians (*6**,**12*). Samples obtained from the cervix, vagina, and vulva all demonstrated HPV DNA, and no one anatomic site accounted for most infections. In one study, HPV DNA was present in 19% of lesbians who reported no previous sex with men (*6*). Importantly, both high- and low-grade squamous intraepithelial lesions (SIL) were detected on Pap smear testing, and they were found in women who reported no previous sex with men. Using capture enzyme-linked immunosorbent assay (ELISA) to measure type-specific antibodies to HPV, we found that 62 (47%) of subjects were seropositive for antibodies to HPV 16, and 83 (62%) were seropositive for HPV 6 (*6**,**13*). Of note, HPV seropositivity among women who reported previous or current sex with men did not differ from that of women who reported no previous sex with men. Given this, and the fact that SIL has been observed in women who reported no previous sex with men, one can conclude that high-risk and low-risk types of genital HPV are sexually transmitted between women and that lesbians should undergo Pap smear screening according to current national guidelines.

Most cases of genital herpes are caused by HSV-2, although recent reports indicate a consistent trend toward more HSV-1–related genital disease (*14*). When Western blot assay was used to detect type-specific antibodies among 392 women in the Seattle Lesbian Health Study, antibodies to HSV-1 were detected in 182 (46%), and to HSV-2 in 31 (8%) (*15*). Most HSV-2 seropositive persons (71%) reported no history of genital herpes, and HSV-1 seroprevalence increased significantly as number of female partners increased. Older age predicted a higher seroprevalence of both HSV types, and HSV-2 seropositivity was associated with a reported history of having had a male partner with genital herpes (but not with number of previous male sex partners). Of 78 women reporting no previous sex with men, 3% were HSV-2 seropositive. Although genital transmission of HSV-2 between female sex partners occurs in a relatively inefficient manner, lesbians' relatively frequent practice of orogenital sex may place them at somewhat higher risk of genital infection with HSV-1, a hypothesis supported by the association between HSV-1 seropositivity and previous number of female partners.

Although *T. pallidum* infection is relatively uncommon, compared to the viral STIs discussed above, sexual transmission between female partners has recently been reported (*16*). Because some lesbians who choose to have sex with men may be more likely to choose bisexual men for partners (*17**,**18*), healthcare providers should keep in mind that the incidence of early syphilis and of fluoroquinolone-resistant *N. gonorrhoeae* have markedly increased in the last several years among men who have sex with men (*19*).

Bacterial vaginosis, a condition associated with depletion of hydrogen peroxide–producing *Lactobacillus* species and the most common cause of vaginitis among women of reproductive age, is associated with pelvic inflammatory disease, increased risk of acquiring gonorrhea and HIV, and adverse outcomes of pregnancy (*20*). The prevalence of bacterial vaginosis among lesbians is high, and vaginal colonization with hydrogen peroxide-producing lactobacilli is low, relative to that of heterosexual women matched for age and sexual risk behavior (*5**,**7**,**21**,**22*).

Bacterial vaginosis prevalence among lesbians in these studies has ranged from 24% to 51%, compared to 21% for heterosexual clients of sexually transmitted disease clinics and 9%–14% for pregnant women. Although bacterial vaginosis is not a classic STI in that a specific microbial precipitant has not been identified, among heterosexual women, report of a new male sex partner and unprotected intercourse are frequently associated (*20*). Moreover, in early studies of "*Hemophilus vaginalis* vaginitis," Criswell and Gardner transmitted bacterial vaginosis from one woman to another by transferring vaginal secretions of women with bacterial vaginosis to noninfected women (*23*). Indeed, bacterial vaginosis is frequently found in both members of monogamous lesbian couples (*7**,**24*). In these women, bacterial vaginosis has been associated with sexual behavior that is likely to result in the transfer of vaginal fluid (*7*). These observations have prompted some authors to propose that sexual transmission of some etiologic factor, as yet undefined, is responsible (*24*).

Finally, lesbians who are also currently sexually active with men may, in some settings, demonstrate increased sexual risk-taking behavior. Among women attending STD clinics, those who report sex with women in addition to sex with men also had a marked increase in HIV-related risk behavior, including sex with gay or bisexual men, use of injection drugs and crack cocaine, and exchange of sex for drugs or money (*17**,**18*). In the 1997 College Alcohol Study, comprised of 14,251 randomly selected U.S. college students, women who reported sex with both men and women were more likely to report multiple sex partners than their peers who had partners of the opposite sex only (*25*). In addition to STI exposure from male partners, previous or current sex with men has obvious implications for lesbians' reproductive health status. The prevalence of reported lifetime pregnancy among lesbians in the studies that have addressed this issue ranged from 23% to 35% (*26**–**28*). Among 392 women in the Seattle Lesbian Health Study, 1 in 4 participants had been pregnant, and >50% had used oral contraceptives (mean duration, 40 months) (*28*). Sixteen percent of all persons and 63% of those previously pregnant reported having at least one induced abortion. The most common pregnancy outcome for women who became pregnant at age <25 years was induced abortion, which occurred in 59% of these pregnancies.

## Preventive Healthcare, Including Pap Smear Screening, in Lesbians

Despite the observations that support probable sexual transmission of HPV between women, many lesbians undergo routine Pap smear screening less frequently than national guidelines advise. In the Seattle Lesbian Health Study, 236 (95%) of respondents believed they should receive Pap smears annually or every 2 years after normal smear results, but 90 (36%) provided a reason for not having done so (*12*). Reasons most commonly cited were lack of insurance, adverse experience at prior Pap smear screening, and a belief they did not need it because they were not sexually active with men. Nine study participants were told (by physicians in all but one case) that they did not need a Pap smear because they were not sexually active with men. Despite high levels of education and income, women with no previous sex with men were less likely to have ever received a pelvic examination, had their first Pap smear at an older age, and had Pap smears less frequently than women who reported previous sex with men. Other investigators have also reported a lower rate of recent pelvic examinations or Pap smears among lesbians (*29**,**30*). Among the few nationally representative surveys, the Boston Lesbian Health Project used snowball sampling (participants from the group of interest are asked to refer members of their social or sexual network for consideration for enrollment in the study) to query a national sample of 1,633 lesbians (*31*). Although the overall screening rates approximated those of the general population, 39% of respondents <20 years and 16% of those 20–29 years had never had a Pap smear, and 29% of those 30–39 years had not had one in >3 years.

One of the few population-based surveys performed with lesbians as the intended audience used a random digit dialing survey to compare the physical and mental health status of 4,135 respondents as a function of self-reported sexual orientation (*32*). Both lesbians and bisexuals were more likely to report increased rates of poor physical and mental health, as other studies have also noted (*33*). Reasons for these findings are unclear. Potential barriers to preventive care by lesbians include healthcare providers' lack of knowledge about disease risk and indications for screening; providers' failure to obtain a complete sexual history from lesbians when relevant, or to do so in a sensitive, nonjudgmental manner; patients' lack of economic resources (due to lack of insurance in the absence of domestic partner benefits, unwillingness to disclose sexual orientation to obtain such benefits when they are offered, or lower earnings in households without at least one man); and lesbians' perception of low risk for STI acquisition from female partners and of cervical dysplasia. Many lesbians (53%–72%) do not disclose their sexual behavior to physicians when they seek care, and disclosures may elicit negative reactions (*34*). Moreover, among 1,086 lesbians surveyed, only 43% of women with a clear risk factor for HIV perceived themselves to be at risk (*35*). Similar assumptions about HPV acquisition from female partners may place lesbians at risk for delayed detection of cervical cancer by less frequent Pap smear screening or none. Finally, lesbians who do not also have sex with men may not access venues providing hormonal contraception, thus eliminating another routine opportunity for Pap smear screening to be sought or offered.

## Conclusions and Future Directions

Available data strongly suggest that HPV, and probably other STIs, are sexually transmitted between women. Thus, recommendations for Pap smear screening among lesbians should not differ from those for heterosexual women, a point that should be clearly communicated in national guidelines and relevant training programs. For example, no national guidelines for STI or Pap smear screening or treatment mention the existence of lesbians, if even to note that data to direct recommendations are limited or absent (*36*). Moreover, healthcare providers, particularly those in training, would benefit from education to enhance their skills in taking a thorough, sensitive sexual history from all patients. The recent increases in STI among men who report sex with men but who do not identify themselves as gay also show that simply asking patients their self-defined sexual orientation is not adequate. Assessment of specific sexual risk behaviors and of previous sexual history can provide a more complete tool for assessment and counseling of patients' sexual health status.

From the research perspective, high-risk HPV types and SIL among lesbians support the need for further investigation. Conditions that could contribute to more infrequent Pap smear screening, including perception of low risk, provider behaviors, or economic barriers, should be defined, as should risk for specific STI transmission. Prevalence of common STI, especially *C. trachomatis* infections, should be systematically studied among young women at highest risk for such infections. Beginning to describe the sexual networks in which lesbians participate—particularly as they involve men at potentially high risk for STI, notably HIV—should provide much-needed data into the sexual and social dynamics of a highly diverse population. The intriguing observation of bacterial vaginosis concordance within female sexual partnerships should offer an opportunity to decipher the puzzling etiology of this common condition. This information could not only contribute to advances in understanding the microbiologic and sociologic characteristics of STIs in general, but more immediately, would inform a cogent approach to counseling lesbians and educating healthcare providers about STI-related risk and prevention.
